# Waist Circumference and Body Mass Index as Predictors of Health Care Costs

**DOI:** 10.1371/journal.pone.0002619

**Published:** 2008-07-09

**Authors:** Betina Højgaard, Dorte Gyrd-Hansen, Kim Rose Olsen, Jes Søgaard, Thorkild I. A. Sørensen

**Affiliations:** 1 Danish Institute for Health Services Research, Copenhagen, Denmark; 2 Institute of Public Health, University of Southern Denmark, Copenhagen, Denmark; 3 Institute of Preventive Medicine, Copenhagen University Hospital, Centre for Health and Society, Copenhagen, Denmark; National Institute for Public Health and the Environment, Netherlands

## Abstract

**Background:**

In the present study we analyze the relationship between body mass index (BMI) and waist circumference (WC) and future health care costs. On the basis of the relation between these anthropometric measures and mortality, we hypothesized that for all levels of BMI increased WC implies added future health care costs (Hypothesis 1) and for given levels of WC increased BMI entails reduced future health care costs (Hypothesis 2). We furthermore assessed whether a combination of the two measures predicts health care costs better than either individual measure.

**Research Methodology/Principal Findings:**

Data were obtained from the Danish prospective cohort study Diet, Cancer and Health. The population includes 15,334 men and 16,506 women 50 to 64 years old recruited in 1996 to 1997. The relationship between future health care costs and BMI and WC in combination was analyzed by use of categorized and continuous analyses. The analysis confirms Hypothesis 1, reflecting that an increased level of abdominal fat for a given BMI gives higher health care costs. Hypothesis 2, that BMI had a protective effect for a given WC, was only confirmed in the continuous analysis and for a subgroup of women (BMI<30 kg/m^2^ and WC <88 cm). The relative magnitude of the estimates supports that the regressions including WC as an explanatory factor provide the best fit to the data.

**Conclusion:**

The study showed that WC for given levels of BMI predicts increased health costs, whereas BMI for given WC did not predict health costs except for a lower cost in non-obese women with normal WC. Combining WC and BMI does not give a better prediction of costs than WC alone.

## Introduction

In epidemiologic research on obesity it is debated whether a combined use of waist circumference (WC) and Body Mass Index (BMI) is a better instrument for identifying high risk individuals [1]; [2]. The reasons for this increased focus are that numerous studies have shown the importance of the body fat distribution as a health risk factor [3], and, secondly, the fact that BMI and WC reflect different body compositions-particularly at the lower end of their distributions. BMI is a proxy measure of total body fat and not fat distribution, whereas WC is a proxy measure of abdominal fat mass [4]. Therefore, the argument has been that a combination of the two measures will represent a better predictor of the variation in health risk.

Bigaard et al. have recently emphasized the importance of using both BMI and WC as a predictor of all cause mortality [1]. Only a few studies have focused on morbidity in general in the investigation of the combined use of BMI and WC in the identification of high-risk individuals, and the available studies focus on one co-morbidity at a time [5]–[10]. This is probably due to the complexity of the problem, which involves a wide range of obesity-related diseases. In addition, previous economic studies of the excess health care costs associated with obesity have applied either BMI or WC as the obesity measures [11]–[19].

The existing economic studies report that health care costs increase with increased obesity status [11]–[19]. In view of the distinct differences in the associations between BMI and mortality and between WC and mortality, when conditioned on each other [1], it would be relevant to investigate whether the combined use of WC and BMI improves the identification of high costs individuals. The mortality associations have their basis in underlying morbidity, but will only partially reflect the overall morbidity problems of obesity, because they involve several health problems not associated with increased mortality, e.g. chronic musculoskeletal disorders.

In the present study we analyze the relationship between future health care costs and the BMI and WC in combination assessed at one point in time. Future health care costs may be interpreted as a measure of the intensity of contacts with the health care sector and may hence be seen as a proxy for morbidity. Clearly, morbidity does not necessarily incur need for health care services if treatments are not available, offered or needed. Also, severity of illness and treatment costs are not always proportional. Nevertheless, one would assume a strong correlation between morbidity and health care consumption. The advantage of applying health care consumption as a measure of morbidity is that it requires no *a priori* definitions of what constitutes obesity related diseases. This measure consequently implicitly constitutes a broad and unconstrained definition of obesity-related morbidity.

We hypothesized that for all levels of BMI, increased WC implies added health care costs (Hypothesis 1). Second, we expected that BMI has a protective effect and that for given levels of WC increased BMI implies reduced health care costs (Hypothesis 2). Third, we assessed whether the prediction of future health care costs was improved by combining the two measures compared to using them separately.

## Methods

### Research Methods and Procedures

Data were obtained from the Danish prospective cohort study Diet, Cancer and Health (DCH) [20]. The population of the DCH study constituted individuals born in Denmark, aged 50–64 years at baseline, residents in the greater Copenhagen or Aarhus areas, and had no records of cancer in the Danish Cancer register at the time of selection [20]. The baseline health status screening was conducted between December 1993 and May 1997 and includes: Anthropometric measurements, blood pressure, collection of biological material, questions on diet and lifestyle. Details of the cohort study have been described previously [20].

A total of 160,725 individuals were invited to participate in the DCH study and of these a total of 55,705 participated in the study. Only individuals included in DCH in the period January 1996 and May 1997 were included in the present study, due to constraints on the accessibility of uniform costs data (reporting procedures varied in the previous time period). Health status, lifestyle and socioeconomic variables were assessed at base-line while individual data on health care consumption and associated costs were extracted for the subsequent 7 years (1996/1997 to 2003/2004).

### Health Care Costs

Only direct public health care costs were included in the analyses. The health care system in Denmark is predominantly publicly financed by income taxes and other taxes, and all public health care services are registered in various registers. All Danish residents have a 10-digit individual identification number (CPR number) which enables identification of resource consumption at the individual level. For each study subject, the following information on health care use was obtained: (I) somatic in- and out-patient treatments (retrieved from the National Patient Register), (II) psychiatric in- and out-patient treatments (retrieved from the Danish Psychiatric central Register [21]) (III) primary sector health care services, including general practitioners, practicing specialists, dentists, physiotherapists, psychologists etc. (retrieved from the National health Insurance Register) and (IV) prescription drugs entitled to a subsidy (retrieved from the national medicine database).

The Danish Case Mix System (Diagnostic related groups; DRG) was used to assign costs to all somatic outpatient and inpatient services. Since DRG charges are only available for somatic services, the National Board of Health's per diem charge and ambulant charge for psychiatric treatments were used to calculate the cost of psychiatric treatment. The cost of primary sector health care services was estimated by using the refunding price plus the patient's costs (for some of the services in the primary sector only a part of the total cost is refunded). The retail cost at the date of purchase was used to assign cost of prescription drugs.

The costs were adjusted to 2005 price level and aggregated at the individual level. The mean annual health care cost per subject was calculated by dividing the total costs by the number of person years registered in the 7 year follow up period. In the following we refer to this figure as future health care costs. The results are presented in US$ (Danish kroner (DKK) and converted to US$ using currency rate 100 DKK = US$17.40).

### Statistical analyses

In order to eliminate potential sources of bias due to confounding factors such as illness related weight loss, the analyses were restricted to individuals with a BMI of 18.50 kg/m^2^ or more and without a history of cancer at baseline. We allow for gender differences in the relationship between health care consumption and BMI and WC status by gender-specific analysis. When analyzing the relationship between future health care costs and BMI and WC these explanatory variables were treated as categorical and continuous variables, respectively.

#### Analysis based on BMI and WC as categorical variables

BMI and WC were cross-tabulated to show the distribution of subjects across the nine evaluated BMI/WC-categories reflecting the nine possible combinations of WHO's three BMI categories (normal weight, overweight and obese) and three WC categories (normal, increased and substantially increased WC) [4]. For definitions of the WC and BMI categories see Table 1.

**Table 1:** WHO's cut points for the classification of obesity status according to Body Mass Index (BMI) and Waist Circumference (WC) (4).

Classification	BMI (kg/m^2^)	
Normal range	18.50–24.99	
Overweight	≥25	
Obese	≥30	
	WC(cm)	
Classification	Women	Men
Normal	<80	<94
Increased	≥80	≥94
Substantially increased	≥88	≥102

To investigate whether the combined use of these measures improves the identification of high costs persons, the annualized mean health care costs were calculated for the nine different combinations of BMI and WC categories. One way ANOVA followed by Bonferroni's post hoc test was used to compare the means.

#### Analysis based on BMI and WC as continuous variables

Ordinary least-squares (OLS) regression was used to analyze the relationship between future health care cost and BMI and WC. The dependent variable was the logarithm of future health care costs. Costs were logged because preliminary examination indicated that these data were characterized by a skewed distribution with a long right hand tail as is frequently observed for health care cost data (after the log conversion: kurtosis = 3.876; skewness = 0.137).

Five Models were applied to investigate the relationship between future health care costs and BMI and WC (separately and combined). Model 1 and 2 test the relationship between future health care costs and WC and BMI. In order to test whether additional information is obtained through a combined use of BMI and WC the interaction term BMI×WC was added to the regressions in Model 3 and 4. In Model 3 the interaction term may potentially capture any systematic impact that BMI status has on the relationship between health care costs and WC. In Model 4 the interaction term represents the possible impact that WC may have on the cost-BMI relationship. There is reason to believe that BMI only has a protective effect in the lower categories of WC and BMI [1]. Therefore, the model specification applied in model 3 was run on the subgroup of individuals with BMI <30 kg/m^2^ and WC <88 cm for women and 102 cm for men (Model 5). The applied obesity measures were all treated as continuous variables.

In order to adjust for potential confounding the following potential explanatory variables were included in all Models: age, income, level of education, smoking status and physical activity (a dichotomous variable indicating none or some sports activities, which in previous studies has proved to be the main predictor of mortality among a broad panel of variables describing the physical activity; Bigaard J et al, unpublished observation). Slope estimates of these potential confounders are not reported in the results in order to simplify the presentation of the key results.

The significance of each partial regression coefficient was assessed using a two-sided Student-t test. Statistical significance was set at *p* = 0.05. Heteroscedasticity was evident and therefore White-corrected standard errors were used [22]. Akaike's information criterion (AIC) was used for parsimonious goodness of fit comparison of the Models. Pearson *χ* ^2^ test was used to assess statistical significance for categorical variables. Statistical analyses were performed using STATA version 9.1.

## Results

A total of 33,083 individuals visited a study clinic in the period January 1996 to May 1997. After exclusion of those with missing values the final sample constituted 31,840 individuals (97%): 15,334 men and 16,506 women. A total of 679 men and 402 women died during the period of observation. The number of deaths amongst men differed significantly across the nine combination categories of WC and BMI (*p*<0.001), while the difference was not statistically significant for the women (*p* = 0.426).The baseline characteristics of the cohort are presented in Table 2.

**Table 2:** Baseline characteristics of the 31,840 eligible participants with complete data (16,506 women and 15,334 men)

	Women			Men's percentiles		
	Median	5 to 95 Percentile	Range	Median	5 to 95 Percentile	Range
Age (years)	56	50 to 64	50 to 65	56	50 to 64	50 to 65
Weight (kg)	67.3	53.3 to 91.1	37.3 to 160	82	65.6 to 105	44 to 151
Height (cm)	164	154.5 to 174	128 to 192	177	166.5 to 188	141.5 to 206
Waist circumference (cm)	80.5	68 to 103	55 to 162	95	82 to 113	63 to 149
BMI (kg/m^2^)	24.9	20.2 to 33.7	18.5 to 57.9	26.2	21.6 to 32.8	18.6 to 50.1

**Figure 1:**
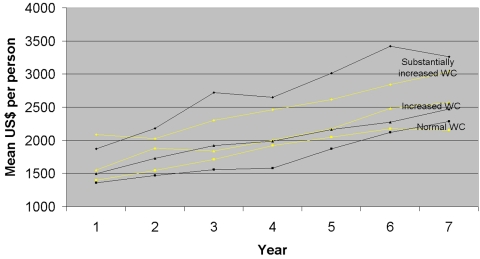
Mean annual health care cost by WC categories (men black, women light).

Figure 1 shows the mean health care cost per year per person for each WC category over a period of seven years. For both genders, the mean health care costs in the year of study entrance were higher for those with substantially increased WC than for those with increased WC (*p*<0.0001). Moreover, individuals with increased WC have higher health care costs than those with normal WC (*p*<0.0001). In general the mean cost per year increase over the study period probably due to ageing of the individuals. However, especially for men, the rise in health care cost is more pronounced for those individuals with substantially increased than for those with normal WC (*p* = 0.0082) and those with increased WC (*p* = 0.0005). Similar trends were observed for BMI categories, but for ease of presentation these are not presented.

### 

#### Analysis based on categorization of BMI and WC

The distribution of the subjects according to the nine combination categories of WC and BMI are shown in Table 3. These distributions show that in women with a normal WC 13.6% will have a higher than normal BMI. Conversely, in women with an increased or a substantially increased WC 36.5% and 6% will have a normal BMI, respectively. In men with a normal WC 30.2% have a higher BMI than normal, and in those who have a normal BMI 15.7% and 1.1% have a increased or a substantially increased WC. The two measurements scales clearly define obese individuals differently. Note that the number of subjects in extreme categories, such as individuals with high BMIs and normal WCs (or vice versa), is very small which reduces our ability to identify statistically significant differences in health care costs across some categories due to lack of statistical power.

**Table 3:** Distribution of the subjects according to the 3 waist circumference (WC) and 3 BMI categories, in combination.

	Normal WC*	Increased WC*	Substantially increased WC*	Total
	No [%]	No [%]	No [%]	
Women				
BMI 18.5 to 24.9 kg/m^2^	6619 [86.3]	1578 [36.5]	269 [6.0]	8466
BMI 25.0 to 29.9 kg/m^2^	1038 [13.5]	2583 [59.8]	2137[47.3]	5758
BMI ≥30.0 kg/m^2^	10 [0.1]	162 [3.7]	2110 [46.7]	2282
Total	7667 [100]	4323 [100]	4516 [100]	16,506
Men				
BMI 18.5 to 24.9 kg/m^2^	4501 [69.8]	778 [15.7]	42 [1.1]	5321
BMI 25.0 to 29.9 kg/m^2^	1944 [30.1]	3968 [80.1]	1840 [46.8]	7752
BMI ≥30.0 kg/m^2^	7 [0.1]	206 [4.2]	2048 [52.1]	2261
Total	6452 [100]	4952 [100]	3930 [100]	15,334

* Definition of the WC categories, see table 1.

Table 4 shows the mean annual health care cost over the 7 year observation period for the three BMI and WC categories, respectively.

**Table 4:** Mean annual health care cost for the different BMI/WC categories (SD)*.

BMI/WC	Total	Normal WC†	Increased WC†	Substantially increased WC†
Women				
BMI 18.5 to 24.9 kg/m^2^, $	2049	1960 (±3255)	2304 (±4808)	2742 (±3577)
BMI 25.0 to 29.9 kg/m^2^, $	2303	1969 (±3480)	2164 (±3349)	2632 (±4206)
BMI > = 30.0 kg/m^2^, $	2607	1097(±609)	2310 (±3562)	2637 (±3404)
Total		1960 (±3284)	2221 (±3950)	2641 (±3813)
Men				
BMI 18.5 to 24.9 kg/m^2^, $	2137	2053 (±4631)	2476 (±4703)	4799 (±6936)
BMI 25.0 to 29.9 kg/m^2^, $	2247	1943 (±4730)	2138 (±3874)	2804 (±4889)
BMI > = 30.0 kg/m^2^, $	3131	2185 (±1770)	2321 (±3625)	3215 (±6124)
Total		2020 (±4659)	2199 (±4007)	3039 (±5595)

* One way ANOVA followed by Boferroni's post hoc test was used to compare the means.

† Definition of the WC categories, see table 1.

No statistically significant differences in costs were found for different levels of BMI within a WC category (lowest *p*-value = 0.246), suggesting that the WC categorization adequately captures the variation in health care costs. In contrast, information on WC appears to capture variation in health care costs within BMI categories. For women with a normal BMI a statistically significant difference in mean cost was found between those with normal WC and increased WC (*p* = 0.025), and for both men and women a statistically significant difference in means was obtained between those with normal WC and substantially increased WC (*p* = 0.018 and *p* = 0.006). In addition, for men and women with 25 kg/m^2^≤BMI <30 kg/m^2^ a significant difference in mean costs was found between those with normal WC and substantially increased WC (*p*<0.001), and between those with increased WC and substantially increased WC (*p*<0.001).

These results indicate that for a given BMI category, information on WC category increases the ability to predict individual's future health care costs, while for a given WC category no additional information was gained by adding the BMI category.

#### Treating BMI and WC as continuous variables

The results of the five regression Models are reported in Table 5. For both men and women a highly statistically significant association between future health care costs and WC (Model 1) and between future health care costs and BMI (Model 2) was found (*p*<0.001). The insignificant estimate of the interaction term in Model 3 suggests that BMI status does not systematically affect the WC-cost relationship. However, in Model 5 (which is equivalent to Model 3 but only includes the subgroup of individuals with BMI<30 kg/m^2^ and WC <88 cm for women and <102 cm for men), the interaction term is associated with a statistically significant coefficient (*p*<0.01) for women. Contrary to Model 3 a statistically significant coefficient of the interaction term was found in Model 4, which demonstrates that inclusion of WC information in addition to BMI status improves the prediction of obesity related health costs.

**Table 5:** The association between health care cost, BMI, WC and BMI×WC. Note that constant term and potential confounders are included in the analysis, but the estimates are not reported

	WC	BMI	WC×BMI	Adjusted R^2^	AIC
Women					
Model 1	0.011***			0.046	46,176
Model 2		0.022***		0.041	46,274
Model 3	0.014***		−0.00005	0.046	46,176
Model 4		−0.054***	0.0005***	0.047	46,162
Model 5†	0.017***		−0.0002**	0.035	
Men					
Model 1	0.017***			0.079	47,672
Model 2		0.043***		0.075	47,738
Model 3	0.013***		0.00007	0.079	47,673
Model 4		−0.056***	0.0006***	0.079	47,667
Model 5†	0.008*		0.00005	0.063	

^*^, ^**^ and ^***^ indicate statistical significance at the 5%, 1% and 0.1% level.

† Model 5 run the model specification applied in model 3 on the subgroup of individuals with BMI<30 kg/m^2^ and WC <88 cm for women and 102 cm for men

In addition, Table 5 shows for both men and women that Model 4 has the lowest AIC score. However, the small difference in AIC values across the regression analysis which includes WC only and the regression analysis which include BMI and WC*BMI is so small that it does not support an argument for the latter. The relative magnitude of the AIC estimates supports the finding that the regressions including WC as an explanatory factor provide the best fit to the data.

Table 5 confirms the findings from the analyses based on categorisation of WC and BMI. Hence, no additional information is gained by a combined use of BMI and WC for a given WC, while for a given BMI the combined use of BMI and WC provides additional information.

## Discussion

The primary objective of this study was to investigate whether the combined use of WC and BMI improves the identification of high costs individuals. The present analysis complements a previous study based on the same dataset which found that a combination of WC and BMI strongly predicted all-cause mortality, in both men and women [1]. Our hypotheses were that: for all levels of BMI, increased WC implies added health care costs (Hypothesis 1) and for a given WC increased BMI implies reduced health care costs (Hypothesis 2).

The results based on the analysis where BMI and WC are treated as continuous variables show that for a given BMI inclusion of WC improves the identification of high costs individuals, reflecting that a more abdominal fat distribution for a given level of BMI gives higher health care costs. The complementary analysis in which BMI and WC are treated as categorical variables suggests the added value of WC is mainly found amongst individual with BMI<30 kg/m^2^, which corresponds to the findings on mortality [1]. Figure 1 demonstrates that future means costs are driven by differences in consumption of health care costs at base-line as well as a difference in the rate of increase in costs over the 7 year observation period.

The previous findings that BMI and WC have opposite effects on total mortality when conditioned on each other [1], that fat mass and lean body mass may have opposite effects on health [23] and that WC captures the deleterious effects of fat mass on mortality [24] have increased the interest in combining these two measurements, thereby potentially achieving an increased accuracy in the identification of individuals at greatest risk. As in many previous analyses, we find a strong positive correlation between WC and BMI (0.87 for men, 0.86 for women). Despite this high correlation between BMI and WC, the results show that the categorization of individuals into risk groups according to their obesity status differs between the two obesity measures. We observe that WC is a more sensitive measure for capturing the high cost individuals. Individuals with a normal BMI but increased or substantially increased WC incur higher mean costs than those with a normal BMI and a normal WC. Interestingly, this group incurs significantly higher mean future health care costs than do individuals who are overweight and obese but have a normal WC.

These results illustrate our general finding that WC is a better predictor of future health care costs. This finding is in agreement with a small study (n = 424) which found that total health care costs were better correlated with WC than with BMI [13]. The findings are also in agreement with recent epidemiological studies which indicate that WC is a stronger marker of health risk than BMI is [25]; [26]. BMI has been criticized for misclassifying muscular subjects as being overweight when, in fact, they are lean [27] and it has been shown that the abdominal fat mass can vary dramatically within a narrow range of BMI [4]. The fact that WC is a good indicator of the location of the excess adipose tissue, and that visceral fat seems to be highly related to health risk [28]; [29] most likely explains this study's observations.

One may, however, argue that the use of WHO's categorization may be suboptimal in this context since the WC and BMI cut-off points were not designed to be used in combination. Rather, the WC cut points were derived by use of BMI [28]. However, as discussed above the categorization of individuals into risk groups according to their obesity status differs between the two obesity measures. Arder et al. [30] have in a recent study shown that the optimal WC thresholds increased across BMI categories when predicting future coronary events, and Bigaard et al. [31] have found the same for total mortality. As also recently discussed by an expert panel [32] these findings indicate that there is a need to investigate whether it is necessary to develop special thresholds when evaluating BMI and WC in combination. Furthermore, it should be noted that despite the widely used recommendation of the applied action levels of WC these are still under debate.

Our results show that BMI coupled with WC did not predict obesity related health risk better than WC did alone. This finding is in agreement with the findings in a previous study which found that BMI coupled with WC did not predict obesity-related health risk (measured by the odds ratios for different metabolic variables, e.g. blood pressure and cholesterol) better than WC did alone when these two anthropometric measures were examined on a continuous scale [26]. However, when WC was dichotomized into normal and high-risk categories, BMI remained a significant predictor of health risk. The authors suggest that the reason for this observation lies in the fact that when WC is treated as a categorical variable whilst BMI is a continuous variable, BMI may capture some of the variation in WC within a WC category. In addition, in a recent article Han et al. conclude that due to the large correlation between BMI and WC, a combination of the two measures adds relatively little to the risk prediction [33].

Recently a panel composed of members with expertise in obesity concluded that the current WC cut points are useless in a clinical practice when BMI is already applied, and that more useful WC cut points are recommended to be found [32]. According to our findings WC rather than BMI should be used.

In this study we have chosen to focus on all types of health care consumption and not only health care consumption related to *an a priori specified* disease associated with obesity such as diabetes and cardio vascular diseases. A specific focus on one disease provides a fragmented description of the disease profile of obese individuals. Instead, we chose the non-discriminatory approach and focused on all types of health care consumption in order to provide a fuller description of the association between obesity status and need for health care services. This strategy introduces a large degree of variation in health care costs that may not be directly related to obesity status. Consequently, the statistical models in table 5 produce R^2^ values in the range 0.04 and 0.08. However, despite this broad perspective and without focus on predefined specific obesity related diseases we are still in position to show that WC is a better predictor than BMI.

Zweifel and colleagues have previously proposed the so-called ‘red herrings’ hypothesis that proximity to death is a more important predictor of health-care costs than is age [34]. Our results support the red herrings hypothesis, as individuals who died in the observation period incurred higher health care costs in the year prior to death. To the extent that persons with high BMI and/or high WC were more at risk of dying, they would on average incur higher future costs. Amongst men, we found a higher mortality amongst the obese (when defined in terms of WC as well as BMI) hence proximity to death may be one of the drivers underlying the observed higher rate of increased health care costs amongst obese men. The differences in mean annual costs may in this case overestimate potential cost savings associated with reduction in obesity.

There are both strengths and limitations to this study. A major strength is the large population sample (n = 31,840), which ensures sufficient power in the analyses. In addition, individual data on health care consumption and associated cost were extracted from valid registers and the anthropometric measurements were measured by trained staff. Potential sources of bias and confounding factors including illness-related weight losses were sought eliminated with the exclusion of subjects with BMI under 18.5 kg/m^2^ or with a history of cancer. Except for the cost data, the analyses were based on cross-sectional data, implying only one single measurement of WC and BMI, leaving information's about the WC and BMI status in the follow-up period missing. This implies that the study results are based on associations of inter-individual WC and BMI differences, and not intra-individual WC and BMI changes on health care costs. Also, there is a risk of selection bias, since only one-third (35%) of the invited individuals participated in this study, and it is likely that in general it is the healthier fraction who chose to participate in the study.

### Conclusions

Our results show that combined use of WC and BMI increases the prediction of high cost individuals, for a given BMI, reflecting that a more abdominal fat distribution for a given BMI gives higher health care costs. However, inclusion of BMI information for a given WC only increases the prediction of future health cost amongst women with BMI<30 kg/m^2^ and WC <88 cm.
